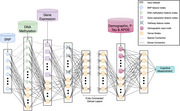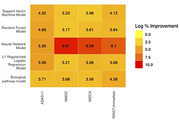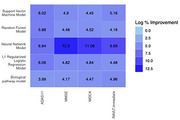# Predicting Individual’s Cognitive Performance Through Multi‐Omics Blood Data Using Hierarchical Input Neural Network ‐ HINN

**DOI:** 10.1002/alz.094598

**Published:** 2025-01-09

**Authors:** Yashu Vashishath, Sarah Beaver, Serdar Bozdag, Fahad Saeed

**Affiliations:** ^1^ University of North Texas, Denton, TX USA; ^2^ BioDiscovery Institute / University of North Texas, Denton, TX USA; ^3^ Florida International University, Miami, FL USA

## Abstract

**Introduction:**

Detecting declines in cognitive function is a critical global health concern, highlighting the need for timely identification to implement effective intervention strategies. This study investigates the potential of blood‐based biomarkers as accurate and non‐invasive measures of cognitive function. We developed a novel deep learning architecture that integrates multi‐omics data by considering their relationship. Specifically, we integrated single nucleotide polymorphisms (SNPs), gene expression, and DNA methylation data to predict cognitive test scores.

**Methods:**

A novel computational architecture called Hierarchical Input Neural Network (HINN) (Figure 1) was developed to integrate multi‐omics datasets. In this study, HINN had three input layers receiving SNPs, DNA Methylation, and gene expression data, respectively. First, we conducted multiple GWAS runs to identify significant SNPs associated with cognitive scores based on different cognitive tests and found 373 SNPs across these runs. We identified 13037 DNA methylation sites proximal to these SNPs and 455 genes associated with these probes.

**Results:**

We observed that MAE of the HINN model for MMSE, MoCA, ADAS11, and RAVLT‐I was 2.28, 2.98, 4.54, and 8.88, respectively. Similarly, MSE of the same model for MMSE, MoCA, ADAS11, and RAVLT‐I was 10.41, 17.16, 45.30, and 135.59, respectively. We compared our model with the other baseline models, namely L1‐regularized, support vector machine, random forest, and deep neural network. We also compared HINN with the pathway guided deep neural network in which all omics data were fed to single layer, which was connected based on biological processes relationship. The HINN model showed improvement of 2.98% to 9.81% for MAE and 3.99% to 12.3% for MSE in comparison to all other models (Figure 2).

**Conclusion:**

The findings derived from the HINN model highlight its efficacy in detecting novel biomarkers for cognitive assessment. Through the hierarchical input approach, we outperformed baseline methods to predict cognitive score. Notably, our method is characterized by its independence from assumptions and subjective scoring, ensuring the robustness and reliability of predictions across diverse cognitive measures. The versatility of our model, capable of predicting multiple cognitive metrics, amplifies its utility and confidence in biological studies targeting cognitive assessment.